# Isoenergetic Pre‐Exercise Meals Varying in Carbohydrate Similarly Affect Resistance Training Volume Performance Compared to Placebo: A Crossover Trial

**DOI:** 10.1002/ejsc.12274

**Published:** 2025-03-03

**Authors:** Andrew King, Ivan Jukic, Colby A. Sousa, Caryn Zinn, Eric R. Helms

**Affiliations:** ^1^ Sport Performance Research Institute New Zealand (SPRINZ) Auckland University of Technology Auckland New Zealand; ^2^ Division of Sport and Exercise Sciences School of Applied Sciences Abertay University Dundee UK; ^3^ Department of Exercise Science and Health Promotion Muscle Physiology Laboratory Florida Atlantic University Boca Raton Florida USA

**Keywords:** appetite, carbohydrate, metabolism, resistance training performance, sport nutrition

## Abstract

Carbohydrate is an important fuel during moderate‐ to high‐intensity exercise. We hypothesised that pre‐exercise carbohydrate ingestion would improve resistance training (RT) volume performance. In a crossover design, sixteen resistance‐trained participants (male = 13 and female = 3) performed 3 sets of back squats, bench press, prone row, and shoulder press to repetition fatigue at 80% of 1‐repetition maximum (∼90 min). Two hours prior, in randomised order, participants ingested high carbohydrate (HCHO; 1.2 g/kg body mass), low carbohydrate (LCHO; 0.3 g/kg body mass), or a low‐calorie placebo (PLA), taste‐ and texture‐matched liquid breakfasts. Linear mixed models were used to analyse volume performance, subjective appetite ratings, and blood glucose and lactate. There were no significant differences between conditions for repetitions completed per session (*p* = 0.318) or exercise (*p* = 0.973). Pre‐exercise and postexercise hunger was similar between conditions (*p* = 0.155). Satiation was greater in HCHO and LCHO versus PLA postbreakfast (*p* = 0.007 and *p* = 0.002, respectively) and pre‐exercise (*p* = 0.001 and *p* = 0.002). Fullness was greater in HCHO and LCHO versus PLA postbreakfast (*p* = 0.001 and *p* = 0.001, respectively) and pre‐exercise (*p* < 0.001 and *p* < 0.001). Blood lactate was greater mid‐ (*p* < 0.001) and postexercise (*p* < 0.0001) and was similar between conditions (*p* = 0.897). Blood glucose significantly increased 30 min after breakfast in HCHO versus LCHO and PLA (*p* < 0.001) and was similar between conditions postexercise (*p* = 1.000). The macronutrient or energy composition of a pre‐exercise meal does not enhance upper‐body‐dominant RT volume.

**Summary**

- When consuming a moderate dietary carbohydrate (≥ 3 g/day), acute carbohydrate intake before a primarily upper‐body resistance training session may not influence volume performance.
- Improved subjective appetite with energy intake may not influence volume performance.
- Blood glucose concentration is maintained during resistance training, even in the absence of any nutrient provision.

## 1 Introduction

Dietary carbohydrate (CHO) is an important fuel source for moderate‐ to high‐intensity exercise, and the provision of CHO before and/or during exercise is recommended by current sport nutrition guidelines (Thomas, Erdman, and Burke 2016; Kerksick et al. 2017). Dietary CHO is stored in the liver and skeletal muscle as glycogen, which is an important energy source for high‐intensity exercise (Vigh‐Larsen et al. 2021). Standard resistance training (RT) volumes induce modest muscle glycogen store decrements (24%–40%) (Koopman et al. 2006; Tesch, Colliander, and Kaiser 1986; Pascoe et al. 1993; Macdougall et al. 1999) and greater RT volumes cause greater decrements (Robergs et al. 1991). Skeletal muscle contains several distinct muscle glycogen depots (e.g., intramyofibrillar, intermyofibrillar and subsarcolemmal) (Ørtenblad et al. 2015). Intramyofibrillar glycogen stores are associated with Ca2+ release from the sarcoplasmic reticulum during contraction, and these stores become selectively depleted after standard RT volumes (Hokken et al. 2020). The combined impact of a modest decrease in total muscle glycogen and the depletion of intramyofibrillar stores may induce fatigue and limit exercise performance during RT (Ørtenblad et al. 2011; Jensen et al. 2020; Nielsen et al. 2009, 2014). In addition, during periods of fasting, such as the overnight fast, liver glycogen becomes progressively depleted, whereas muscle glycogen stores remain stable (Knapik et al. 1988; Rothman et al. 1991). Nonetheless, CHO feeding can modestly increase muscle glycogen stores (10%–15%) after an overnight fast (Wee et al. 2005; Chryssanthopoulos et al. 2004), and acute CHO feeding before RT may augment glycogen stores in preparation for RT.

Although there are recommendations for peri‐workout nutrition for endurance training that intend to augment glycogen storage, there is less evidence to establish RT‐specific recommendations. Acute CHO ingestion improved RT volume performance, as reported in a recent meta‐analysis (King et al. 2022), where the pre‐exercise fast and session duration were longer (≥ 8 h and ≥ 45 min, respectively), and the ergogenic effect of CHO was greater as more volume was completed compared to low‐ or zero‐energy placebo. In addition, several recent trials reported the potential impact of acute CHO feeding on RT volume performance via psychological (i.e., expectancy) and appetite suppression mechanisms (Naharudin et al. 2020, 2021).

No previous trial investigating the effect of acute CHO feeding on RT performance has used a comparator placebo condition while equating energy intake within CHO conditions. When energy is not matched between conditions, an ergogenic effect of CHO feeding may be due to energy provision itself rather than any specific metabolic influence of CHO on performance. In this sense, energy provision in general could provide varying fuel sources for exercise, a psychological effect (e.g., placebo), or an effect on subjective appetite not necessarily exclusive to CHO that could affect RT volume performance. Thus, we designed a randomised, double‐blind crossover trial investigating the effect of two isoenergetic and isonitrogenous pre‐exercise meals with higher (1.2 g/kg body mass) and lower (0.3 g/kg body mass) CHO content and a low‐calorie placebo on volume performance during a high‐volume, full‐body, yet ecologically valid RT session. Given the established role of CHO in exercise performance, we hypothesised that HCHO would improve volume performance compared to LCHO and PLA and that LCHO would improve performance compared to PLA only.

## 2 Materials and Methods

### 2.1 Participants

Using previously published data for session squat repetitions completed (M. Naharudin et al. 2020), an a priori power analysis was completed using Power Analysis and Sample Size (PASS; version 15.0.5) software, which revealed that 15 participants would be required to achieve a power of 0.8, an effect size of *f* = 0.28, an alpha *α* = 0.05, and a correlation *ρ* = 0.7. The correlation assumption was checked by a statistician separate from data collection after the first six participants had completed all trials, which was robust (*ρ* = 0.78).

**FIGURE 1:**
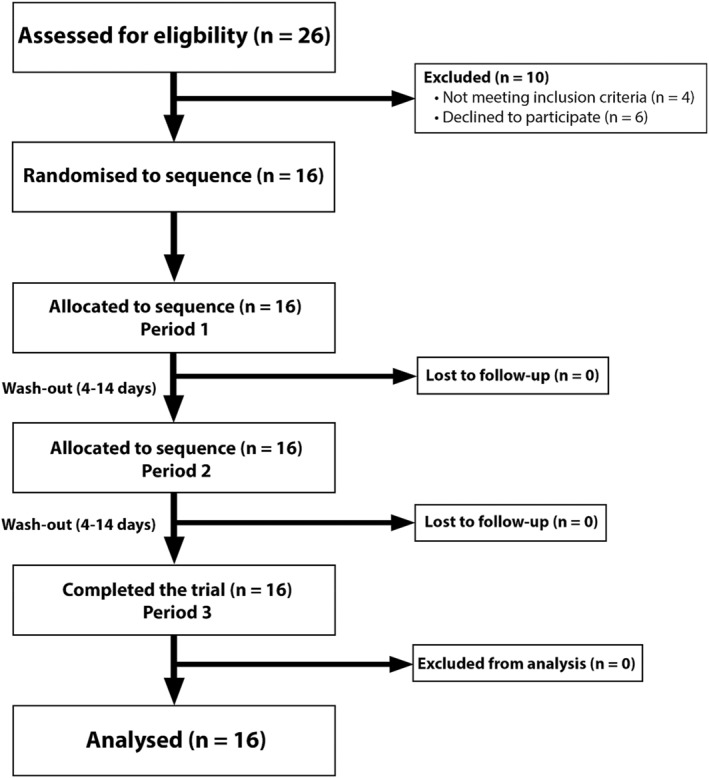
CONSORT participant flow diagram through each stage of the randomised crossover trial.

Resistance‐trained males (*n* = 13) and females (*n* = 3) were recruited via advertisement (i.e., posters, social media, and university courses). Their descriptive characteristics are presented in Table 1. Twenty‐six participants were initially screened for participation, of whom *n* = 16 were deemed eligible and volunteered to participate in the trial. All participants provided written consent before commencing this study and completed testing with no dropouts. The CONSORT participant flow diagram is presented in Figure 1. The study protocol was approved by the University Ethics Committee (20/312). To be eligible for inclusion, potential participants must have been (a) able to squat 1.5 and 1.25× bodyweight for males and females, respectively; (b) able to bench press 1.0 and 0.75× bodyweight for males and females, respectively; (c) between 18 and 40 years in age; (d) a habitual consumer of breakfast (> 5 times per week); (e) not have a pre‐existing injury, metabolic disease or condition or medical condition that would contraindicate safe participation in exercise; (f) not reportedly using ergogenic insulin‐like substances and/or anabolic/catabolic steroids, prohormones, or hormones known to affect muscle mass; (g) not reporting dietary requirements contraindicating the ingestion of a breakfast (e.g., reactions or aversions to ingredients); and (h) not previously privy to the contents of the pre‐exercise meals (i.e., volunteers who pilot tested were aware of the nutritional composition).

**TABLE 1:** Participant descriptive characteristics (*n* = 16).

	Participants
Age (years)	26 ± 4
Height (cm)	176.6 ± 7.5
Body mass (kg)	83.68 ± 15.1
Resistance training experience (years)	4.8 ± 2.24
Relative squat strength (1RM/body mass)	1.81 ± 0.4
Relative bench press strength (1RM/body mass)	1.26 ± 0.3

*Note:* Data are presented as mean ± standard deviation. 1RM = 1‐repetition maximum.

### 2.2 Design

This study was a double‐blind, randomised, counterbalanced, crossover trial consisting of, in order, two familiarisation sessions, one 1‐repetition maximum session, and three experimental high‐volume RT sessions. For each experimental session, participants arrived in the morning after an overnight fast (> 10 h) and consumed one of three pre‐exercise liquid breakfasts, which were taste, texture, colour, and volume matched in pilot testing in a randomised order: (A) high CHO (HCHO; 1.2 g/kg body mass CHO), (B) an isoenergetic and isonitrogenous low CHO (LCHO; 0.3 g/kg body mass CHO) or (C) a low‐calorie placebo (PLA) liquid breakfast. HCHO and LCHO primarily contained vanilla Super Mass Gainer (Dymatize, NC, U.S.) and vanilla KetoMeal (KetoLogic, NC, U.S.), respectively. HCHO and LCHO also contained water (350–500 mL), butter powder (Garden of Life, FL, U.S.), and pure maltodextrin (NZ Starch, Auckland, NZ), in varying amounts depending on body mass. PLA contained water, guar gum (Ceres Organics, Auckland, NZ), and vanilla FlavDrops (MyProtein, Manchester, UK). The sample average liquid breakfast nutrition values are presented in Table 2. Participants were told during familiarisation that the three conditions contained the same amount of total energy but differed in macronutrient composition. After the third experimental session was completed, participants learnt of the true study design and were asked if they could confidently identify which trial was PLA.

**TABLE 2:** Average nutritional content of pre‐exercise breakfasts (*n* = 16).

	High CHO (1.2 g/kg BM)	Low CHO (0.3 g/kg BM)	Placebo
Total energy (kJ)	2438 ± 478	2412 ± 505	11 ± 0
Relative total energy (kJ/BM)	29 ± 1	29 ± 1	0.1 ± 0
CHO (g)	103 ± 20	25 ± 5	0 ± 0
Protein (g)	20 ± 5	21 ± 4	0 ± 0
Fats (g)	10 ± 2	44 ± 9	0 ± 0
Water intake (mL)	1699 ± 608	1653 ± 719	1805 ± 747

Abbreviations: BM = body mass; CHO = carbohydrate.

Two hours after consuming the liquid breakfast, participants completed three sets of back squat, bench press, prone row, and shoulder press with 80% 1‐repetition maximum (1RM), with repetitions completed to fatigue. Participants were randomised according to the Latin square method. Possible sequences (i.e., ABC, ACB, BCA, BAC, CAB, and CBA) were placed in individual sealed, opaque envelopes. A researcher independent of all data collection kept the sealed envelopes in a locked drawer and, during participant sign‐up, selected an envelope, assigned the sequence to the participant, and then discarded the sequence. Once all six possible sequence combinations were assigned, a new block of six envelopes was generated. All testing was completed between April 2021 and May 2022. This trial was not prospectively preregistered on a public registry.

### 2.3 Familiarisation (Visits 1 and 2)

Participants' height (cm) and weight (kg) were recorded using a stadiometer (Seca Ltd, Hamburg, Germany) and a digital calibrated scale (Tanita HD366, Tanita Corporation, Tokyo, Japan). Participants were verbally informed of the testing protocol for the 1RM and experimental sessions. Participants were familiarised with the RT‐specific RPE/repetitions in reserve (Zourdos et al. 2016) and subjective appetite (i.e., hunger, satiety, and fullness) visual analogue (Flint et al. 2000) scales, and the instruction to lift as fast as possible during the concentric phases (with a self‐selected eccentric tempo), which was implemented for all sessions, with feedback after each set during familiarisation. Participants were also instructed to take at least a momentary pause, but no longer than 2 s, between repetitions. Participants were asked what their best, most recent set of each exercise (or closest variation) was, to be used with the Lombardi prediction equation (Lombardi 1989) to estimate a conservative 1‐repetition maximum for 1RM testing.

Participants performed a standardised dynamic warm‐up and completed sets of 5, 3, and 1 repetition with 20%, 40%, and 60% of the estimated 1RM in the back squat, bench press, prone row, and shoulder press, with a self‐selected rest. After a mandatory 3‐min rest, participants completed 10 repetitions with 60% of the estimated 1RM. A 20‐kg barbell (Rogue, Columbus, Ohio, USA) and calibrated weight plates (Viking, Wellington, NZ) were used in all sessions. The squat and bench press were performed in accordance with International Powerlifting Federation regulations using only approved “unequipped” lifting material aids (i.e., knee sleeves and a weightlifting belt). Briefly, the back squat required a depth at which the hip crease passed the top of the knee when viewed laterally. For the bench press, the necessary contact points were maintained (i.e., head, upper back, buttocks and flat feet). The prone row and seated shoulder press were performed as previously outlined (Spiering et al. 2009). For the prone row, the chest had to maintain contact with the bench, and the barbell had to touch the bottom of the bench, during which participants always wore lifting straps (VersaGrips, Maine, USA). The seated shoulder press was performed off safety pins in a squat rack and required the buttocks and upper back to remain in contact with the chair throughout the movement. A successful seated shoulder press repetition required the participant to raise the barbell off the safety pins to full elbow extension overhead before controlling the eccentric movement of the barbell back onto the safety pins. All participants completed two familiarisation sessions, which were separated by at least 72 h.

### 2.4 Pretrial Standardisation

Participants were asked to track and record their daily food intake using the MyFitnessPal (various versions) app. During the two familiarisation sessions, the participants were provided with a digital food scale, downloaded MyFitnessPal, practised inputting various food selections into MyFitnessPal, and received verbal instructions from a researcher on best practices for weighing, measuring, and recording daily food intake. Participants were instructed to consume a daily CHO (4–7 g/kg BM) and protein (at least 1.6 g/kg BM) intake in accordance with current sport nutrition recommendations for RT athletes (Morton et al. 2018; Slater et al. 2013) during participation in this study. Participants were not instructed regarding food selection but were provided with food suggestions, where necessary, to enable adherence to the daily carbohydrate and protein intakes. In addition, participants were instructed not to intentionally increase or decrease their total daily energy intake and to maintain their current supplement habits (i.e., not to introduce or stop taking supplements and to maintain the dose). When the participant was habituated to pre‐workout supplementation, they were asked to consume the same dose of supplement at the same time point prior to all sessions in this study. This was coordinated with a researcher and required the supplement to be low in total energy (<10 kcal). Participants were required to meet the daily CHO and protein intake recommendations for at least 3 days preceding each experimental trial, which was verified by a researcher who had access to the participants' MyFitnessPal logs. Participants’ water intake was not quantified during the pretrial period. Participants were asked to maintain their normal training habits between experimental trials and to refrain from physical activity for 48 h preceding 1RM and experimental trials. Experimental sessions were separated by at least 4 days and up to 10 days, to provide adequate recovery time and to meet the standardised pretrial nutrition requirements between experimental sessions.

### 2.5 1RM (Visit 3)

The 1RM protocol consisted of 3 repetitions at 20%, 40%, and 60%; 1 repetition at 80% and 90%, followed by up to five 1RM attempts (Jukic et al. 2020a, 2020b). Mean concentric velocity, as measured by a linear position transducer (Gymaware Kinetic Performance, Canberra, Australia), was used in concert with participant‐reported RPE/RIR to guide attempt selection. If a 1RM attempt was successful, the load was increased by 1–12.5 kg in consultation with the participant. A 1RM was recorded if the participant successfully completed the lift at 10 RPE or successfully completed an attempt at a lower RPE but failed a subsequent attempt. Three minutes and 3‐ to 5‐min rest were given between submaximal sets and 1RM attempts, respectively. Barbell velocity feedback was not provided to participants during the 1RM session or at the subsequent experimental sessions. Constant verbal encouragement was provided by researchers. The 1RM for all four exercises was completed on the same day.

### 2.6 Experimental Trials (Visits 4–6)

After an overnight fast (> 10 h), participants arrived at the laboratory at the time they habitually ate breakfast (all participants arrived between 0700 and 0900). The selected start time was kept consistent for each participant. Upon arrival, participants verbally confirmed adherence to the overnight fast (> 10 h) and abstinence from physical activity (48 h); then, body mass was recorded. Thereafter, participants consumed the pre‐exercise meal within 15 min, which was prepared and delivered in an opaque container by a researcher independent of all data collection. To aid with blinding, participants were asked not to look in the opaque container and were required to wear a nose peg during its ingestion and for 2‐min following. A 10‐s water mouth rinse was performed after ingestion, which was expectorated. Participants remained in the seated position for 2 h after completing the meal. Water was provided *ad libitum* throughout each experimental trial, which was measured and recorded by a researcher.

Two hours after the pre‐exercise meal, participants began the RT session. Participants completed the same standardised warm‐up before completing 3 sets of back squat, bench press, prone row, and seated shoulder press, with repetitions completed to 10 RPE (i.e., no repetitions left in reserve) at 80% of 1RM. Exercise order was consistent for all participants and experimental trials. Rest before and between 10 RPE sets was 3 min. Rest between exercises was 5 min. Each exercise was preceded by exercise‐specific warm‐ups of 5, 3, and 1 repetition with 50%, 70%, and 90% of the working load, with 60‐ to 90‐s rest. Music was permitted at the discretion of the participant, and the same playlist and volume were used for all experimental sessions. Participants were reminded by researchers to complete the concentric muscle action as fast as they could for all lifts, and standardised verbal encouragement was given to participants throughout all experimental sessions.

Performance outcomes of interest were total session repetitions completed and total repetitions completed per exercise, which were silently counted by a researcher. Secondary outcomes were blood glucose and lactate and subjective appetite (i.e., hunger, satiety, and fullness). The researcher who recorded all performance, metabolic, and subjective appetite values did not provide verbal encouragement to the participant.

Blood glucose was recorded at baseline (PREbreakfast), 30 min (POST30 min) and 60 min (POST60 min) after the liquid meal; and before (POST120 min) and after (POST210 min) the RT session. Blood lactate was recorded immediately before (PREexercise), midway (MIDexercise; after bench press) and at completion (POSTexercise) of the RT session. Capillary blood glucose and lactate were drawn and analysed using a glucose (StatStrip Xpress2, Nova Biomedical, Germany) and lactate (Lactate Pro 2 LT‐1730, Arkray, Japan) metre, respectively; and recorded by a researcher who was not providing verbal encouragement.

**FIGURE 2:**
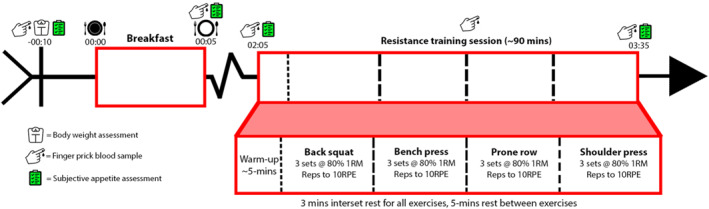
Experimental session overview. Note that the times are approximations. 1RM = 1‐repetition maximum and RPE = the resistance training repetitions in reserve/rating of perceived exertion scale.

Subjective appetite (hunger, satiety, and fullness) was recorded before and after breakfast (PREbreakfast and POSTbreakfast, respectively) and the RT session (PREexercise and POSTexercise, respectively). An overview of the experimental session is illustrated in Figure 2.

### 2.7 Statistical Analyses

All data were normally distributed as determined by graphical inspection and conventional value ranges for skewness and kurtosis (Gravetter et al. 2020; Field, Miles, and Field 2012; Trochim et al. 2001). Descriptive data are presented as mean ± standard deviation. Outcomes of interest were the total number of repetitions completed per set and session; perceptual ratings of hunger, satiety, and fullness; and measures of blood glucose and lactate. To examine the effect of condition (i.e., HCHO, LCHO, and PLA) on the outcome of interest, linear mixed‐effects models were used. Condition (3 levels) and exercise (four levels) were treated as fixed effects, and participants were treated as random effects. Random slopes were introduced to the models when their inclusion did not result in a convergence error. Given the incorporation of both fixed and random effects, restricted maximum likelihood estimation was used to fit the models. Hedges' *g* effect sizes were calculated, and the magnitude of the difference was determined by standard thresholds: small (0.2–0.49), moderate (0.5–0.79) and large (> 0.8) (Cohen 1988).

Multicollinearity was checked by inspecting the variance inflation factors for all predictor parameters included in the linear mixed‐effects model. The independence of observations was confirmed by performing autocorrelation diagnostics. For all linear models, a Gaussian distribution was assumed. Goodness‐of‐fit was checked by assessing the approximate normal distribution of model residuals. Plotted residuals were checked to ensure homoscedasticity before applying the results of the model to ensure all assumptions were met.

All statistical analyses were conducted in R language and environment for statistical computing (R Core Team 2021) using the *lme4* (Bates et al. 2015), *emmeans* (Lenth et al. 2018), and *ggeffects* (ggeffects 2018) packages. Model assumptions were checked using the *performance* (Lüdecke et al. 2021) and *DHARMa* (Hartig 2020) packages. The custom‐written R script and associated dataset are available on the Open Science Framework repository (URL: https://osf.io/sc2up/). The figures in this manuscript present condition (i.e., HCHO, LCHO, and PLA) means with 95% confidence intervals; however, if the reader is interested in individual responses across conditions, these are available on the repository.

## 3 Results

### 3.1 Baseline Nutrition, Session Duration, and Breakfast Perception

Average 3‐day protein (*p* = 0.545), fat (*p* = 0.929), CHO (*p* = 0.739) and total energy (*p* = 0.843) intake between conditions produced a statistically nonsignificant result.

The average session duration was 93.4 ± 5.2 min. *Ad libitum* water intake during the experimental trials produced a statistically nonsignificant result between conditions (*p* = 0.783).

Nine of the 16 participants (56%) correctly identified which breakfast was PLA. Six stated that they could not identify which breakfast was PLA. One participant stated they could identify which breakfast was PLA but was incorrect.

### 3.2 Performance Outcomes

There was no significant main effect of trial (Trial 2: *p* = 0.57 and Trial 3: *p* = 0.06 compared to Trial 1, respectively) or interaction between condition and trial (*p* = 0.35–0.84).

**FIGURE 3:**
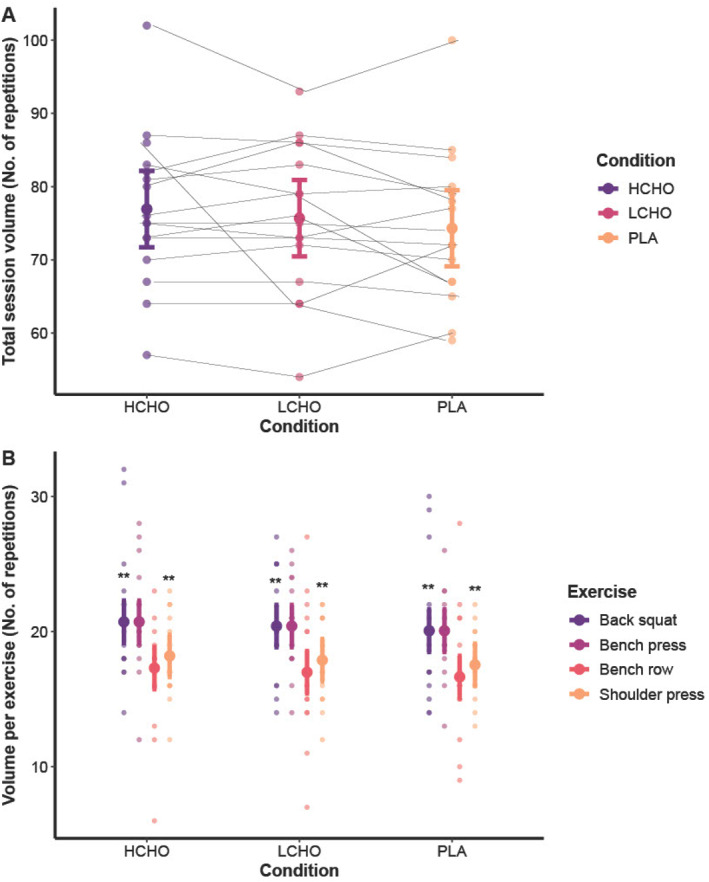
Comparison between groups (HCHO, LCHO and PLA) for total session repetitions completed (Panel A) and repetitions completed per exercise (Panel B) after a high‐volume resistance training session, as well as individual data points. Data were analysed using linear mixed models and are presented as mean and 95% confidence intervals. HCHO = high carbohydrate breakfast, LCHO = low carbohydrate breakfast, PLA = placebo, SQ = back squat, BP = bench press, and SP = shoulder press. ** significantly greater than prone row and shoulder press (*p* ≤ 0.001).

For total session repetitions completed, there was a nonsignificant result between conditions (*F* = 1.192; *p* = 0.318). For total session repetitions completed per exercise, there was a nonsignificant interaction between condition and exercise (*F* = 0.208; *p* = 0.973). There was a main effect of exercise on total session repetitions completed (*F* = 6.84; *p* < 0.001) as more squat repetitions were completed per session than prone row (*p* < 0.001; *g* = 0.63) and shoulder press (*p* = 0.001; *g* = 0.5). More bench press repetitions were completed per session than prone row (*p* < 0.001; *g* = 0.83) and shoulder press (*p* = 0.001; *g* = 0.66). Performance outcomes are presented in Figure 3.

### 3.3 Subjective Appetite

#### 3.3.1 Hunger

**FIGURE 4:**
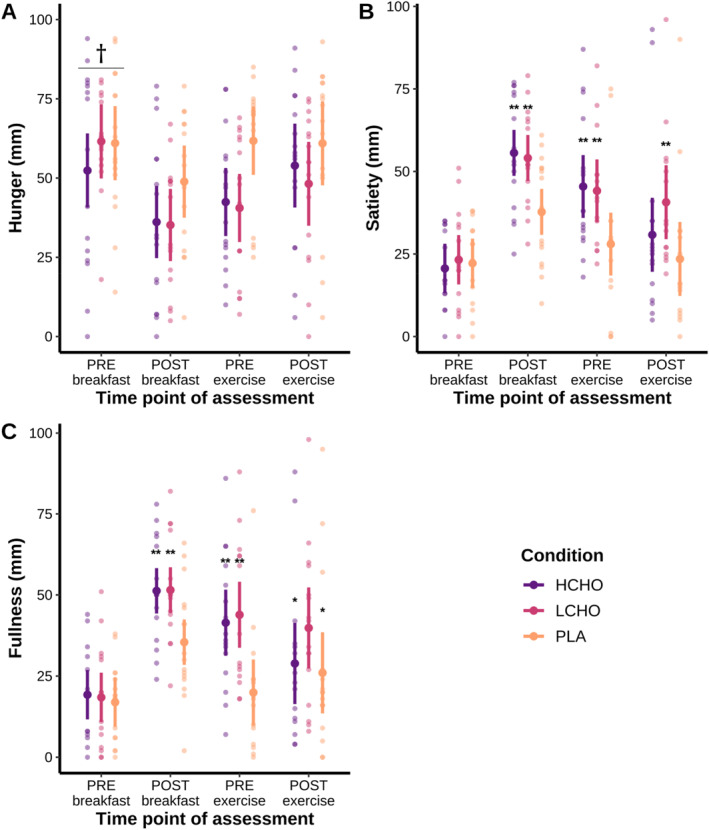
Comparison between groups for subjective hunger (Panel A), satiety (Panel B), and fullness (Panel C) ratings after a high‐volume resistance training session, as well as individual data points. Data were analysed using linear mixed models and are presented as mean and 95% confidence intervals. HCHO = high carbohydrate breakfast, LCHO = low carbohydrate breakfast and PLA = placebo. † significantly greater than POSTbreakfast (*p* < 0.01); ** significantly greater than PLA (*p* ≤ 0.001); and * significantly lower than LCHO (*p* ≤ 0.05).

There was a nonsignificant interaction between condition and time point (*F* = 1.594, *p* = 0.155). There was a significant main effect of time on hunger (*F* = 3.37, *p* = 0.027), as PREbreakfast was significantly greater than POSTbreakfast (*p* = 0.002). The results for hunger are presented in Figure 4A.

#### 3.3.2 Satiety

There was a significant interaction between condition and time point (*F* = 2.625, *p* = 0.02). Compared to PLA, satiety at the POSTbreakfast time point was significantly greater in HCHO (*p* = 0.001, *g* = 0.68) and LCHO (*p* = 0.002, *g* = 0.69). Compared to PLA, satiety at the PREexercise time point was significantly greater in HCHO (*p* = 0.001, *g* = 0.78) and LCHO (*p* = 0.002, *g* = 0.71). Compared to PLA, satiety at the POSTexercise time point was significantly greater in LCHO (*p* = 0.001, *g* = 1.15), but not in HCHO (*p* = 0.076, *g* = 0.37). The results for satiety are presented in Figure 4B.

#### 3.3.3 Fullness

There was a significant interaction between condition and time point (*F* = 3.672, *p* = 0.002). Compared to PLA, POSTbreakfast fullness was significantly greater in HCHO (*p* = 0.001, *g* = 0.63) and LCHO (*p* = 0.001, *g* = 0.63). Compared to PLA, PREexercise fullness was significantly greater in HCHO (*p* < 0.001, *g* = 1.13) and LCHO (*p* < 0.001, *g* = 1.33). POSTexercise fullness was significantly greater in LCHO when compared to HCHO (*p* = 0.026, *g* = 0.53) and PLA (*p* = 0.005, *g* = 0.72). The results for fullness are presented in Figure 4C.

### 3.4 Metabolic Markers

#### 3.4.1 Lactate

**FIGURE 5:**
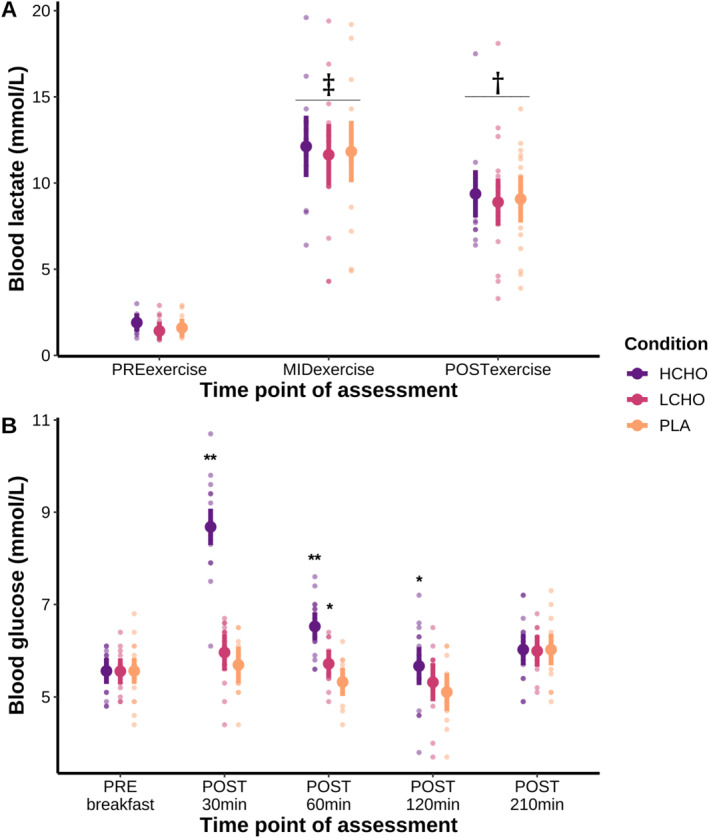
Comparison between groups for blood lactate (Panel A) and glucose (Panel B) concentrations after a high‐volume resistance training session, as well as individual data points. Data were analysed using linear mixed models and are presented as mean and 95% confidence intervals. HCHO = high carbohydrate breakfast, LCHO = low carbohydrate breakfast and PLA = placebo. † significantly greater than PREexercise (*p* < 0.001); ‡ significantly greater than PREexercise (*p* < 0.001) and POSTexercise (*p* < 0.001); ** significantly greater than PLA and LCHO (*p* ≤ 0.001); and * significantly greater than PLA (*p* ≤ 0.05).

There was a significant main effect of time for blood lactate (*F* = 51.03, *p* < 0.001). Blood lactate at MIDexercise was significantly greater than PREexercise (*p* < 0.001, *g* = 2.58) and POSTexercise (*p* < 0.001, *g* = 1.05). Blood lactate at POSTexercise was significantly greater than PREexercise (*p* < 0.001, *g* = 2.27). The results for blood lactate concentration are presented in Figure 5A.

#### 3.4.2 Glucose

There was a significant interaction effect between condition and time point (*F* = 31.595, *p* < 0.001). HCHO blood glucose was higher than LCHO at POST30 min (*p* < 0.001, *g* = 2.46) and POST60 min (*p* < 0.001, *g* = 1.35), and higher than PLA at POST30 min (*p* < 0.001, *g* = 3.11), POST60 min (*p* < 0.001, *g* = 1.94) and POST120 min (*p* = 0.004, *g* = 0.59). LCHO blood glucose was higher than PLA at POST60 min (*p* = 0.025, *g* = 0.27). The results for blood glucose concentration are presented in Figure 5B.

## 4 Discussion

The purpose of this study was to investigate the effect of higher and lower doses of pre‐exercise CHO ingestion on RT volume performance in an ecologically valid exercise protocol while controlling for total calories consumed. The main findings were as follows: (a) RT volume performance was similar between two isoenergetic, isonitrogenous pre‐exercise meals differing in CHO dose and a low‐calorie PLA, (b) participants were generally more full and sated in HCHO and LCHO compared to PLA, (c) pre‐exercise blood glucose was significantly higher in HCHO compared to PLA, but not LCHO, and postexercise blood glucose was similar between conditions, and (d) mid‐exercise and postexercise blood lactate increased compared to pre‐exercise, with no interaction effect between conditions at any time point.

The findings do not confirm our initial hypothesis. We hypothesised that a higher dose of CHO would improve volume performance over a lower dose (LCHO) and a low‐calorie PLA due to the established role of dietary CHO as an important fuel source during high‐intensity exercise (Vigh‐Larsen et al. 2021). RT induces modest decreases in total muscle glycogen (24%–40%) (Robergs et al. 1991), and stores of intramyofibrillar glycogen in Type II muscle fibres can become depleted during RT (Hokken et al. 2020). In some circumstances, CHO can be ergogenic for RT volume performance, such as when the training duration is longer (> 45 min) and the pre‐exercise fast is longer (> 8 h) (King et al. 2022). Given the longer duration (∼90 min) and higher volume performed to repetition fatigue (12 sets) in the RT session of this study, we hypothesised an ergogenic effect of CHO ingestion. However, we found no significant effect of higher or lower CHO doses on RT volume performance compared to a low‐calorie PLA. Possible explanations for the lack of observed results may include exercise selection, training intensity, and the timing of CHO ingestion.

In this study, three of the four exercises were for the upper body (i.e., bench press, prone row, and shoulder press), which were selected as a representative variety of exercises common to strength and hypertrophy‐type training. Previously, King et al. (2022) reported that acute CHO ingestion had a clearer ergogenic effect on higher‐volume lower‐body RT and speculated that this trend may be due to lower‐body RT recruiting more muscle mass, producing more total work, and incurring greater metabolic fatigue. However, few studies have investigated the effects of acute CHO ingestion on higher‐volume upper‐body RT. Krings et al. (2016) reported an ergogenic effect of CHO ingestion on bench press repetitions completed to fatigue compared to a placebo, but no significant performance improvement in the bent‐over row, incline press or close‐grip press. In addition, Smith et al. (2018) reported no significant effect of a liquid CHO beverage on repetitions to fatigue in a ∼60 min upper‐body RT session, nor did the studies by Naharudin et al. (2020) and Bin Naharudin et al. (2019) report an ergogenic effect of pre‐exercise CHO on bench press volume performance compared to water only or a viscous, energy‐less placebo. Overall, the findings from this study and previous literature suggest that CHO ingestion might be less important for volume performance in a higher‐volume upper‐body RT session, potentially due to recruiting relatively less muscle mass than lower‐body RT, resulting in less total work and metabolic fatigue. Future research is necessary to investigate whether isoenergetic pre‐exercise meals similarly affect higher‐volume lower‐body RT.

It has previously been reported that hunger may influence RT performance. Naharudin et al. (2020) reported no significant difference in repetitions to fatigue during 4 sets of back squat and bench press after viscous, semi‐solid CHO‐containing and placebo pre‐exercise meals. However, the CHO and placebo meals in Naharudin et al. (2020) did improve volume performance compared to water only, and the authors concluded that this may be due to psychological mechanisms (i.e., a placebo effect of ingesting or nocebo effect of omitting and a pre‐exercise meal). Indeed, the participants in that study (Naharudin et al. 2020) were hungrier and less full in the water‐only control compared to the taste‐ and texture‐matched CHO and placebo groups and were similarly hungry and full following CHO and placebo. The findings of this study are broadly in agreement with Naharudin et al. (2020). In a follow up study (Naharudin et al. 2021), a viscous CHO‐containing breakfast improved subjective appetite and repetitions performed to fatigue compared to a liquid CHO breakfast, suggesting hunger can influence RT performance. In this study, there were no significant differences in hunger between conditions at the pre‐exercise and postexercise time points. However, HCHO and LCHO had higher ratings of fullness and satiety at the pre‐exercise time point, and satiety at the postexercise time point, compared to PLA, indicating that the participants were generally less full and sated after ingesting PLA, compared to HCHO and LCHO. A greater magnitude in the difference of hunger/fullness between conditions in Naharudin et al. (2020) may explain the differences in results compared to this study. Nonetheless, the results from this study suggest that lower subjective appetite may not always influence RT performance and that greater feelings of hunger may be requisite for pre‐exercise feeding to influence volume performance. The discrepancy in results between our study and previous studies (Naharudin et al. 2020, 2021) may be due to differences in the magnitude of effect on subjective appetite (i.e., the previous studies elicited greater differences in appetite between conditions) and exercise protocol, such as training duration, exercise selection (i.e., upper vs. lower) and training intensity (i.e., %1RM load), and the addition of pre‐exercise supplement practices.

Training intensity (i.e., percentage of 1RM) could have also affected the ability to detect an effect of CHO feeding in this study. We selected a load of 80% 1RM to lend ecological validity to the design, which resulted in an average of ∼7 repetitions being completed in each set of each exercise. An increase of 1 repetition would be a ∼14% increase in volume performance, and although our power analysis was based on a 16% difference in volume performance, it is possible that differences in the study design led to nonsignificant results in volume performance. Several other studies using lower training intensities (approximately 55%–75% 1RM) and lower‐body exercise selection have reported an ergogenic effect of carbohydrate compared with water (Naharudin et al. 2020; Bin Naharudin et al. 2019) and an energy‐less placebo (Haff et al. 1999, 2000; Lambert et al. 1991). Thus, it is possible that lower loads and higher repetitions may be required to detect an effect of pre‐exercise feeding, although a recent meta‐regression did not find an effect of load on the ergogenic effect of CHO (King et al. 2022).

Participants were told that all three breakfast meals contained the same amount of energy (despite PLA containing almost no calories). The breakfasts were taste‐ and texture‐matched during pilot testing. Nine of the 16 participants were able to correctly identify the PLA breakfast, which indicates modest success in masking the true design of this study from the participants. Positive expectancy from ingesting a pre‐exercise breakfast perceived to contain energy may explain why there were no significant differences in volume performance in this study. However, without a control condition that omitted the ingestion of anything except water, it is not possible to determine the magnitude of a placebo effect. Nonetheless, previous studies (Naharudin et al. 2020; Bin Naharudin et al. 2019) also indicate that psychological factors (i.e., positive or negative expectancy) of feeding may play a role in mediating RT volume performance.

Another potential mediating factor that could explain the lack of influence of subjective appetite on performance in this study is that half of the participants (*n* = 8) used their habitual pre‐exercise supplements, which could be sufficient to mask an effect of the pre‐exercise meal or to suppress/mitigate sensations of hunger. These supplements included caffeine (black coffee, *n* = 4; pre‐workout formulation, *n* = 4), which has known ergogenic effects on indices of muscle strength and endurance (Grgic et al. 2019) without clear evidence for an effect on appetite (Schubert et al. 2017). Future research should investigate whether habituation to pre‐exercise supplementation mediates the effect of a pre‐exercise meal on RT performance.

There are several limitations to this study. We detected modest evidence that there was an order effect such that more repetitions were completed in Trial 3 than in Trial 1 (*g* = 0.86, *p* = 0.06). An effect of trial order could mask a treatment effect. However, the study design included randomisation with counterbalancing to address a potential influence of a trial effect, and there was no evidence that condition was systematically influenced by trial number (*p* = 0.35–0.8). The sample size calculation for this study was based on detecting a moderate effect between a CHO and water‐only condition for squat repetitions reported in a previous study (Naharudin et al. 2020). Thus, although the sample size of this study is powered to detect an effect between HCHO and PLA conditions, it might be underpowered to detect a small effect between HCHO and LCHO conditions, if one exists. Commercially available products were used to prepare pre‐exercise meals, and their nutritional composition was not verified. Several aspects of our study design could have influenced the results (e.g., supplementation and music) and masked the potential effect of a pre‐exercise meal on RT volume performance. This adds external validity to our findings, because these aids are used in practice, and although they were kept consistent for each participant across trials, their inclusion prevents the true isolation of the effect of the pre‐exercise meal on RT performance. A mixed‐sex cohort of participants was recruited in this study, and it is currently unknown if there are potential sex differences in the response to a pre‐exercise meal on RT volume performance. Finally, we attempted to provide metabolic insight with blood measures but did not measure muscle glycogen; thus, future research is necessary to understand the effect of CHO ingestion on muscle glycogen (and its subcellular compartments) during RT.

## 5 Conclusion

The results provide evidence that for primarily upper‐body RT volume performance, a higher or lower CHO dose produces comparable performance to a low‐calorie placebo. These findings are of practical relevance, as they suggest that the macronutrient composition of a meal may not matter per se, and that the perception of energy intake may be sufficient for RT volume performance (at least in the context of similar session lengths, volumes, and exercise selections).

## Conflicts of Interest

The authors declare no conflicts of interest.

## Author Declarations

The authors have nothing to report.

**Abbreviations**
CHO: Carbohydrate
HCHO: High carbohydrate
LCHO: Low carbohydrate
PLA: Placebo
RT: resistance training
1RM: 1‐repetition maximum

## Data Availability

The custom‐written R script and associated dataset are available on the Open Science Framework repository (URL: https://osf.io/sc2up/).
